# Human convalescent plasma protects K18-hACE2 mice against severe respiratory disease

**DOI:** 10.1099/jgv.0.001599

**Published:** 2021-05-07

**Authors:** Joseph W. Golden, Xiankun Zeng, Curtis R. Cline, Aura R. Garrison, Lauren E. White, Collin J. Fitzpatrick, Steven A. Kwilas, Philip A. Bowling, Jimmy O. Fiallos, Joshua L. Moore, Willie B. Sifford, Keersten M. Ricks, Eric M. Mucker, Jeffrey M. Smith, Jay W. Hooper

**Affiliations:** ^1^​ Virology Division, United States Army Medical Research Institute of Infectious Diseases, Fort Detrick, MD 21702, USA; ^2^​ Pathology, United States Army Medical Research Institute of Infectious Diseases, Fort Detrick, MD 21702, USA; ^3^​ Veterinary Medicine Division, United States Army Medical Research Institute of Infectious Diseases, Fort Detrick, MD 21702, USA; ^4^​ Diagnostic Services Division, United States Army Medical Research Institute of Infectious Diseases, Fort Detrick, MD 21702, USA

**Keywords:** animal models, K18-hACE2 mouse model, SARS-CoV-2, antibody protection, therapeutics

## Abstract

SARS-CoV-2 is the causative agent of COVID-19 and human infections have resulted in a global health emergency. Small animal models that reproduce key elements of SARS-CoV-2 human infections are needed to rigorously screen candidate drugs to mitigate severe disease and prevent the spread of SARS-CoV-2. We and others have reported that transgenic mice expressing the human angiotensin-converting enzyme 2 (hACE2) viral receptor under the control of the Keratin 18 (K18) promoter develop severe and lethal respiratory disease subsequent to SARS-CoV-2 intranasal challenge. Here we report that some infected mice that survive challenge have residual pulmonary damages and persistent brain infection on day 28 post-infection despite the presence of anti-SARS-COV-2 neutralizing antibodies. Because of the hypersensitivity of K18-hACE2 mice to SARS-CoV-2 and the propensity of virus to infect the brain, we sought to determine if anti-infective biologics could protect against disease in this model system. We demonstrate that anti-SARS-CoV-2 human convalescent plasma protects K18-hACE2 against severe disease. All control mice succumbed to disease by day 7; however, all treated mice survived infection without observable signs of disease. In marked contrast to control mice, viral antigen and lesions were reduced or absent from lungs and absent in brains of antibody-treated mice. Our findings support the use of K18-hACE2 mice for protective efficacy studies of anti-SARS-CoV-2 medical countermeasures (MCMs). They also support the use of this system to study SARS-CoV-2 persistence and host recovery.

## Introduction

SARS-CoV-2 is a betacoronavirus that emerged in late 2019 and is the causative agent of COVID-19 [1, 2]. COVID-19 is primarily a human respiratory disease with a wide spectrum of severity ranging from a fever and mild cough, to the development of hypoxia, that can lead to life-threatening acute respiratory distress syndrome (ARDS) [3, 4]. Risk factors for the development of severe disease are higher in the aged and in those with underlying health conditions such as obesity, hypertension and cardiovascular disorders [5, 6]. More severe disease is associated with increased inflammatory cytokine production, damage to the vasculature and coagulopathies (pulmonary emboli and stroke) [7–11]. Neurological disease such as headache, anosmia, ataxia, meningitis, seizures and impaired consciousness are widely reported and may lead to longer term sequela in convalescing patients [12–14]. In some SARS-CoV-2 survivors, long-term sequelae, including fatigue, respiratory problems and brain fog can persist for many months [15–17]. As of 31 March 2021, SARS-CoV-2 has infected 128 540 982 people with 2 808 308 deaths [18]. There is an urgent need for medical countermeasures to prevent this disease or limit disease severity in a post-exposure setting.

SARS-CoV-2 binds to target cells via an interaction between the 139 kDa viral spike protein and the host angiotensin-converting enzyme 2 (ACE2) protein [19–22]. SARS-CoV-2 exploits ACE2 for target cell entry and, as a consequence, host tropism and disease susceptibility is greatly influenced by affinity between these two molecules. While SARS-CoV-2 binds human and hamster ACE2 efficiently, it interacts poorly with murine ACE2 [23] and consequentially, mice are highly refractory to infection [24–26]. We and others [25, 27, 28] reported that mice expressing the human ACE2 protein under the control of the keratin 18 promotor (K18-hACE mice) are highly susceptible to SARS-CoV-2, similar to SARS-CoV [29]. These mice develop a severe respiratory disease and depending on viral dose, succumb to disease. SARS-CoV-2 infection of K18-hACE2 mice also leads to brain infection, characterized by neuron infection and vasculitis. While other mouse models exist that exploit exogenous expression of hACE2 either by transgene expression or transduction by viral vectors [26, 30–32], only K18-hACE2 mice develop a consistent severe and lethal respiratory disease following SARS-CoV-2 intranasal challenge. Other animal models, including Syrian hamsters, develop a mild-moderate, transient lung infection that is rapidly cleared, unless animals are immunosuppressed [33, 34]. Additionally, NHPs only develop a mild, transient respiratory infection by SARS-CoV-2 [35]. Thus, the K18-hACE2 mouse system is the only highly lethal SARS-CoV-2 model using naturally circulating virus. This model has potential utility for the screening and evaluation of early stage medical countermeasures (MCMs) against SARS-CoV-2.

In addition to severe damage to the lungs, SARS-CoV-2 infects the brains of K18-hACE2 mice and causes neuroinflammation including meningitis, vasculitis, encephalitis, damage to neurons and anosmia [25, 27, 36]. Accordingly, the K18-hACE2 model may be useful in the study of neurological infection.

Here, we further explored the K18-hACE2 mouse model for SARS-CoV-2 pathogenesis and evaluated the utility of this model as a means to evaluate candidate MCMs. We show that K18-hACE2 mice that survived SARS-CoV2 infection, after >15 % wt loss, developed persistent brain infection despite the presence of neutralizing antibodies. Additionally, surviving mice had varying degrees of lingering pulmonary damages including inflammation. We also demonstrated that human convalescent plasma (HCP) targeting SARS-CoV-2 can dramatically reduce pathology and protect mice against lethal infection. Overall, our findings support the use of the K18-hACE2 mouse model for the study of SARS-CoV-2 MCM development and the study of pathogenesis, including pulmonary complications and persistent brain infection during convalescence.

## Methods

### Viruses and cells

A third passage SARS-CoV-2 strain WA-1/2020 viral stock was obtained from the CDC and is from a human non-fatal case isolated in January 2020. A master stock of virus was propagated as described previously [25]. Virus was quantified by plaque assay and determined to be endotoxin free. All virus work was handled in BSL-3 containment at USAMRIID.

### Mice

C57BL/6 mice and K18-hACE2 mice [B6.Cg-Tg(K18-HACE2)2Prlmn/J] (6–8 weeks old) were provided by the Jackson Laboratory. Mice challenged intranasally with 2×10^4^ p.f.u. of SARS-CoV-2 WA1 strain 2020 diluted in a total volume of 50 µl of PBS (25 µl per nare). All mice were uniquely identified with numerical ear tags (Stoelting). Anti-SARS-CoV-2 antibody or PBS was injected by the intraperitoneal route on the indicated days in a total volume of 0.5 ml per dose. The plasma was screened for SARS-CoV-2 antibody prior to use in animals. Sick animals were supplied nutrient gel.

### Quantitative reverse transcription PCR

Pharyngeal swabs were taken from anesthetized mice using a sterile 2 mm polyester tipped applicator (Puritan). The tip of the applicator was placed in 250 µl of Eagles modified medium with 5 % Foetal bovine serum, 1 % Pen/Strep, 0.1 % Gentamycin and 0.2 % Fungizone. Then 100 µl of this was added to 300 µl of Trizol LS (ThermoFisher) for RNA extraction. Lung tissue was homogenized in 750 µl Trizol reagent using a gentleMACS dissociator system on the RNA setting. RNA was extracted from Trizol per manufacturer’s protocol. A Nanodrop 8000 was used to determine RNA concentration, which was then raised to 100 ng µl^−1^ in UltraPure distilled water. Total nucleic acid was purified using the EZ1 Virus Mini Kit v 2.0 (Qiagen) according to the manufacturer’s recommendations. Samples were run in duplicate on a BioRad CFX thermal cycler using TaqPath 1-step Quantitative reverse transcription PCR (RT-qPCR) master mix according to the CDC’s recommended protocol of 25 °C for 2 min, 50 °C for 15 min, 95 °C for 2 min, followed by 45 cycles of, 95 °C for 3 s and 55 °C for 30 s. The forward and reverse primer and probe sequences are: 2019-nCoV_N2-F, 5′-TTA CAA ACA TTG GCC GCA AA-3′, 2019-nCoV_N2-R, 5′-GCG CGA CAT TCC GAA GAA-3′, and 2019-nCoV_N2-P, 5′-ACA ATT TCC CCC AGC GCT TCA G-3′. The limit of detection for this assay is 50 copies.

### Plaque reduction and neutralization test

Plaque reduction and neutralization tests (PRNTs) were performed as previously reported [34]. PRNT50 and 80 titres are the reciprocal of the highest dilution that results in a 50 % or 80 % reduction in the number of plaques relative to the number of plaques visualized in the medium alone wells.

### Pseuodotype vesicular stomatitis virus neutralization assay

The pseudotype vesicular stomatitis virus (VSV) neutralization assay (PsVNA) used to detect neutralizing antibodies in sera utilized a non-replicating VSV-based luciferase expressing system described previously [37]. Where indicated 5 % human complement (Cedarlane) was included in the virus: antibody mixture.

### SARS-CoV-2 MAGPIX multiplex SARS-CoV-2 immunoassay

Plasma samples were diluted at 1 : 100 PBS with 0.02 % Tween-20 (PBST) with 5 % skim milk (PBST-SK) (Invitrogen). Recombinant SARS-CoV-2 full trimeric spike was a gift from Dr. Jason McLellan; UT-Austin), S1 (Sino Biological, 40591-V08H), receptor binding domain (RBD) (Sino Biological, 40592-V08H), NP (Native Antigen Company, REC31812-100), and SARS-CoV-1 full spike (Protein Sciences) proteins were coupled to Magplex microspheres using the Luminex xMAP antibody coupling kit (Luminex) according to the manufacturer’s instructions. Each individual antigen-coupled bead were mixed 1 : 1 prior to diluting in PBST to 5×10^4^ microspheres ml^−1^ and added to the wells of a Costar polystyrene 96-well plate at 50 µl per well (2500 microspheres of each antigen bead set per well). The plate was placed on a magnetic plate separator (Luminex) and buffer was removed from the plate. Then, 50 µl of the diluted plasma samples were added to appropriate wells. The plate was incubated with shaking for 1 h at RT, then washed three times with 100 µl of PBST. A 50 µl aliquot of a 1 : 100 dilution of goat anti-human IgA (Abcam, ab99909), mouse anti-human IgM phycoerythrin conjugate (Invitrogen, MA1-10381) or goat anti-human IgG phycoerythrin conjugate (Sigma, P9170) in PBST-SK was added to the wells. The plate was incubated with shaking for 1 h at RT. After incubation, the plate was washed three times and the Magplex microspheres were resuspended in 100 µl of PBST for analysis on the Magpix instrument. Raw data was reported as median fluorescence intensity for each bead set in the multiplex. Assay controls consistent of normal human serum and human serum targeting SARS-CoV-2 (BEI Resources).

### Histology

Tissues were immersed in 10 % neutral buffered formalin for 14 days and cut at 5–6 µM on a rotary microtome, mounted onto glass slides and stained with hematoxylin and eosin (H and E). Examination of the tissue was performed by a board-certified veterinary pathologist.

### *In situ* hybridization

SARS-CoV-2 ISH was performed as described previously [25, 34].

### Immunofluorescence assay

Formalin-fixed paraffin embedded (FFPE) tissue sections were deparaffinized using xylene and a series of ethanol washes. After 0.1 % Sudan black B (Millipore Sigma, Burlington, MA, USA) treatment to eliminate the autofluorescence background, the sections were heated in Tris-EDTA buffer (10 mM Tris Base, 1 mM EDTA Solution, 0.05 % Tween 20, pH 9.0) for 15 min to reverse formaldehyde crosslinks. After rinses with PBS (pH 7.4), the sections were blocked with PBT (PBS+0.1 % Tween-20) containing 5 % normal goat serum overnight at 4 °C. Then the sections were incubated with primary antibodies: rabbit polyclonal anti-myeloperoxidase (MPO) at a dilution of 1 : 200 (A039829-2, Dako Agilent Pathology Solutions, Carpinteria, CA, USA), rat monoclonal anti-CD45 antibody at a dilution of 1 : 100 (05–1416, Millipore Sigma, Burlington, MA, USA), rabbit polyclonal anti-CD68 at a dilution of 1 : 200 (ab125212, Abcam, Cambridge, MA, USA), and mouse monoclonal anti-Ki67 at a dilution of 1 : 200 (clone B56, BD Biosciences, San Jose, CA, USA) or rabbit polyclonal anti-CD31 at a dilution of 1 : 100 (ab28364, Abcam, Cambridge, MA, USA) for 2 h at room temperature. After rinses with PBS, the sections were incubated with secondary goat anti-rabbit Alexa Fluor 488 at dilution of 1 : 500 (Thermo Fisher Scientific, Waltham, MA, USA) and goat anti-mouse or anti-rat Alexa Fluor 568 at a dilution of 1 : 500 (ThermoFisher,) antibodies, for 1 h at room temperature. Sections were cover slipped using the Vectashield mounting medium with DAPI (Vector Laboratories, Burlingame, CA, USA). Images were captured on a Zeiss LSM 880 confocal system (Zeiss, Oberkochen, Germany) and processed using ImageJ software (National Institutes of Health, Bethesda, MD). To quantify the MPO^+^ polymorphonuclear cells, CD45^+^ leukocytes, CD68^+^ macrophages, and Ki-67^+^ proliferating cells, fluorescence-labelled cells were counted in random 1.25×10^5^ µm^2^ area of the stained tissues using ImageJ. To quantify the perivascular CD45^+^ leukocytes, fluorescence-labelled cells were counted in the area within 100 µm radius to CD31^+^ blood vessel. Counted fluorescence-labelled cells were analysed using *t*-test.

## Results

### Pulmonary recovery and brain viral persistence in convalescing mouse survivors

**Fig. 1.**
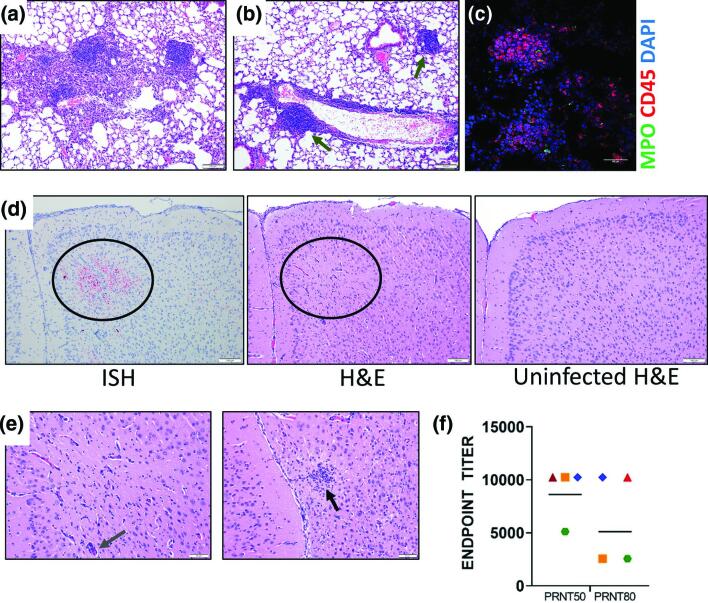
Characterization of K18-hACE2 mouse survivors. (a) H and E staining of mouse lungs showing a focal area of histiocyte infiltration within alveoli and foci of hyperplastic lymphoid tissue from animals that survived SARS-CoV-2 challenge (day 28) (blue arrow). (**b**) Lung from a surviving mouse showing infiltration of lymphocytes surrounding an intermediate sized vessel, and foci of hyperplastic lymphoid tissue (green arrows). (**c**) IFA of lung with hyperplastic lymphoid tissue were stained with CD45 and MPO. Cells were counterstained with DAPI to visualized nuclei.** (d**) Representative ISH staining of infected K18-hACE2 (day 28) mouse demonstrating presence of viral RNA in the cerebral cortex. H and E staining of this same area (circle) shows minimal gliosis and perivascular infiltration of mononuclear leukocytes compared to an uninfected mouse brain in the same region. (**e**) H and E staining from the same mouse in D showing a focal glial nodule (black arrow) in the rostral cerebral cortex in the image on the right, and a higher magnification of the centre image in panel D showing minimal gliosis and perivascular infiltrates (slate arrow).** (f**) Mouse serum (*n*=4 survivors) was evaluated for its ability to neutralize SARS-CoV-2 by plaque reduction assay. Endpoint PRNT_50_ and PRNT_80_ titres were plotted from eleven different replicates. The geometric mean titre are plotted, each shape/colour represents an individual mouse (*n*=4). The limit of quantitation was 40.

SARS-CoV-2 pathogenesis studies in animal models have mainly focused on acute infection of SARS-CoV-2 and not recovery. To understand the pathology underlying pulmonary function abnormalities in COVID-19 survivors [38, 39], we extended the in-life portion of mice (*n*=4) that survived SARS-CoV-2 strain WA01/2020 induced disease after a period of marked weight loss (>15 %) for signs of disease to 28 days post-infection [25]. Hyperplasia was seen within the bronchus-associated lymphoid tissue (BALT) and lymphoid tissue adjacent to vessels in the lungs of all four surviving mice along with perivascular infiltration of mononuclear inflammatory cells. (Fig. 1a, b). Additionally, focal infiltration by histiocytes was detected in alveoli of one of four surviving mice (Fig. 1a). The lymphoid tissue with hyperplasia contained CD45^+^ immune cells (Figs 1 and S1c, available in the online version of this article). An increase of CD68^+^ cells (macrophages) was detected in surviving mouse lungs (average 46.3 CD68^+^ macrophages per 1.25×10^5^ µm^2^ tissue area, *P*<0.01, *n*=9 areas) in comparison with uninfected mouse lungs (average 16.2 CD68^+^ macrophages, *n*=13 areas, Fig. S1a), however, there was no increase in Ki-67 (proliferating cells, 9.9 Ki-67^+^ cells per 1.25×10^5^ µm^2^ tissue area, *P*>0.05, *n*=9 areas, Fig. S1a), CD45^+^ (leukocytes, 35.8 cellsper 1.25×10^5^ µm^2^ tissue area, *P*>0.05, *n*=10 areas, Fig. S1b), and MPO (granulocytes, 17.4 MPO^+^ cells per 1.25×10^5^ µm^2^ tissue area, *P*>0.05, *n*=10 areas, Fig. S1b) staining in comparison with uninfected mouse lungs **(**11.2 Ki-67^+^ cells, 33 CD45^+^ cells, and 16.2 MPO^+^ cells per 1.25×10^5^ µm^2^ tissue area, *n*=13, 13, and 13 areas, respectively, Fig. S1a, b). SARS-CoV-2 genomic RNA was undetectable by ISH in the lungs of any day 28 surviving animal. Within the brain of a single day 28 surviving mouse, viral genome was detected in the rostral section of the cerebral cortex, corresponding with an area of minimal gliosis and perivascular infiltration of mononuclear leukocytes (Fig. 1d, e). A SARS-CoV-2 plaque reduction and neutralization assay revealed that all four surviving mice, including the mouse with persistent brain lesions, had neutralizing antibody with a mean 50 and 80% neutralization titre of 8960 and 6400, respectively (Fig. 1f **)**. These findings indicate that mice surviving SARS-CoV-2 infection can have unresolved brain lesions despite the presence of neutralizing antibodies at least 28 days after exposure.

### Characterization of human convalescent plasma targeting SARS-CoV-2

**Fig. 2.**
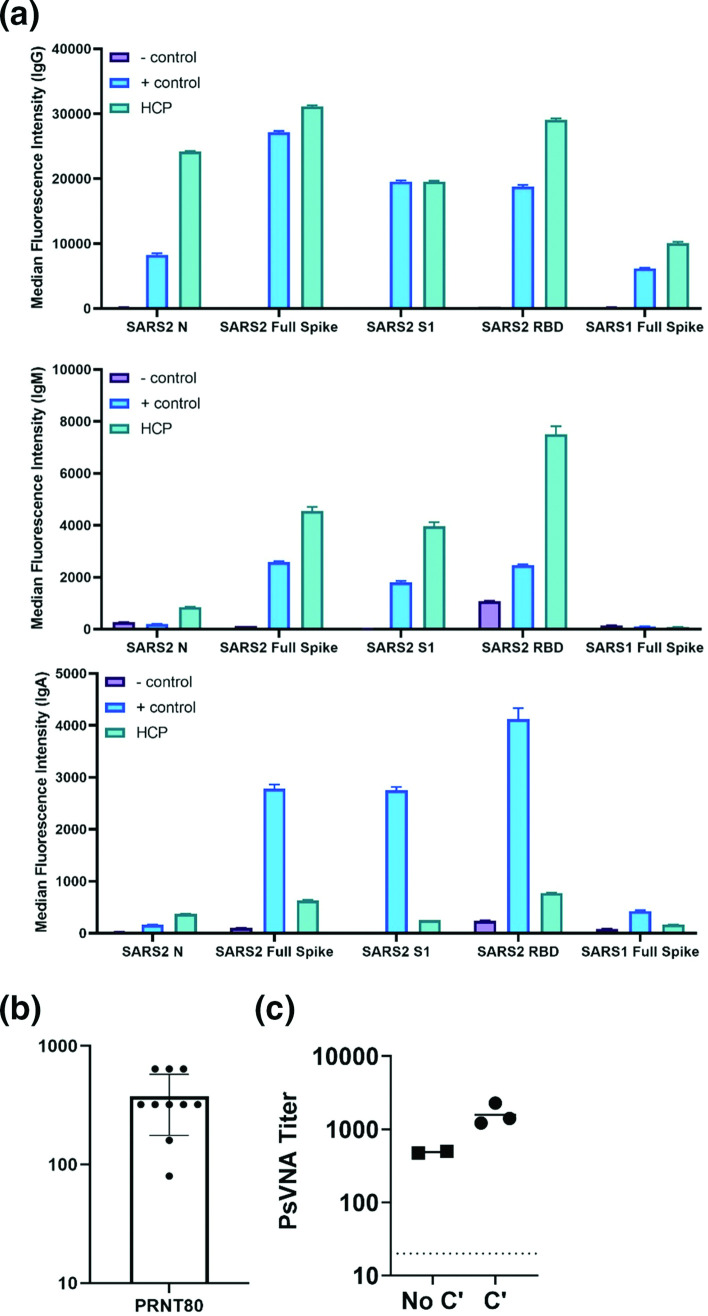
HCP binds SARS-CoV-2 antigen and neutralizes infection. (a) The ability of HCP to bind the indicated SARS-CoV-2 antigens were determined by a multiplex Magpix assay as described in the methods. Normal human serum and serum against SARS-CoV-2 was used as the negative (-) control and positive (+) controls. (**b**) Neutralization activity of anti-SARS-CoV-2 convalescent human plasma was evaluated for by plaque reduction assay. Geometric mean PRNT_80_ titres were plotted from eleven different replicates. The geometric mean titre and SEM are plotted. The limit of quantitation was 40. (**c**) PSVNA50 and 80 titres were determined. The dashed line indicates limit of detection.

To establish the utility of the K18-hACE2 model for screening medical countermeasures, we evaluated the ability of HCP to limit severe disease. Prior to animal studies, plasma from an individual SARS-CoV-2 infected survivor was characterized for its ability to bind N protein and spike protein. Using a multiplex bead assay, we found that the sample of HCP had reactive IgM, IgG and IgA antibodies that bound to both N and S proteins (Fig. 2a **)**. The highest values were for IgG antibodies. We also found that HCP bound the S1 domain of the S protein, along with the receptor binding domain (RBD). Limited cross interaction with SARS-CoV was observed with the IgG component of the HCP. Normal human serum and human serum against SARS-CoV-2 were used as negative and positive controls in the multiplex assay. Using a wild-type virus neutralization assay, we determined that the HCP had an 80 % neutralization geometric mean titre of 320 against strain WA01/2020 (Fig. 2b), and an 80 % titre of 488 against VSV particles pseudotyped with SARS-CoV-2 spike protein (Fig. 2c). Addition of 5 % complement enhanced neutralizing by 3.3-fold (Fig. 2c). These data showed that a single batch of human plasma from a SARS-CoV-2 survivor had antibody targeting the N and spike protein, with neutralization activity.

### Anti-SARS-CoV-2 human convalescent plasma protects K18-hACE2 mice against lethal viral challenge

**Fig. 3.**
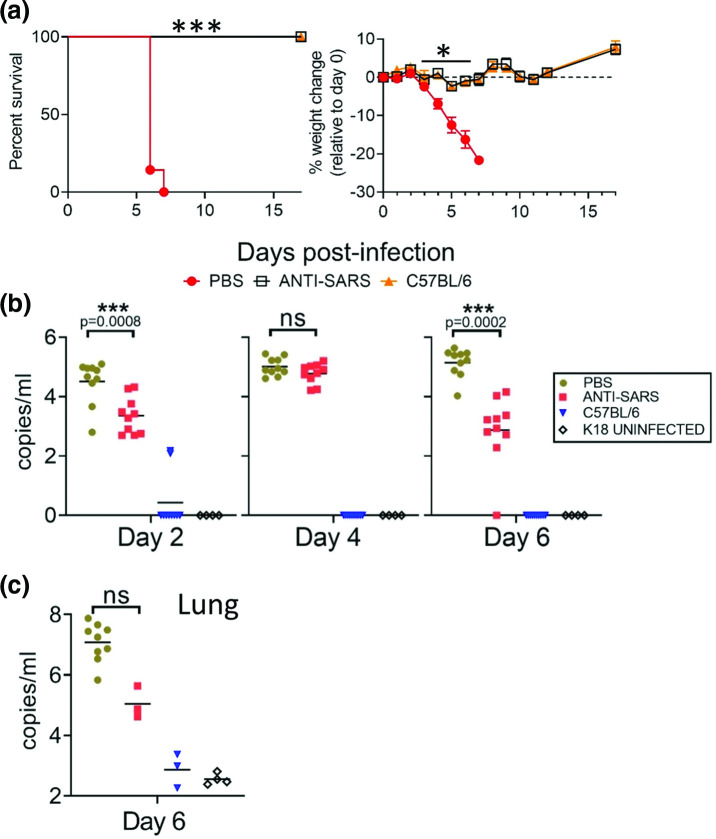
HCP protects against lethal infection in K18-Ace2 mice. (a) K18-Ace2 transgenic mice and C57BL/6 mice (Day 0–3 *n*=10 per group; Day 6+, *n*=7) were infected with SARS-CoV-2 by the IN route. Survival and weight loss were monitored for 17 days and plotted using Prism software. Survival significance determined by log-rank; ***P=0.0001, weight loss significance at the indicated time points was determined by *t*-test; *P<0.005. (**b**) Pharyngeal swabs qPCR titres were taken on the indicated time points. Line shows geometric mean titres graphed as copies ml^−1^. Significance was determined by *t*-test and denoted by asterisks with indicated p value. (**c**) Lung qPCR titres were examined on day 6 (*n*=3 mice per group) or at the time of euthanasia by qRT-PCR (PBS-treated mice *n*=7 mice). Mean titres+/-SEM of the genome copies ml^−1^ were graphed. Significance was determined by *t*-test. NS=not significant.

We evaluated the protective efficacy of the SARS-CoV-2 targeting HCP in K18-hACE2 and C57BL/6 mice. Two groups of 9 week old K18-hACE2 mice and one group of C57BL/6 mice (*n*=10 per group; 50 % male/female) were infected intranasally with 2×10^4^ p.f.u. of SARS-CoV-2 strain WA01/2020. HCP or PBS was administered intraperitoneally on day −1 and +2 at a dose of 0.5 ml per mouse per dose. The control (PBS-treated) group of infected K18-hACE2 mice began to lose weight on day 3 (Fig. 3a). Weight loss was comparatively reduced in HCP-treated mice, with maximal mean weight loss of 2.32 % compared to 21.69 % for PBS-treated mice. Starting on day 6, most PBS-treated K18-hACE2 animals began to show signs of respiratory disease, to include laboured breathing, and lethargy. On day 6 and 7 all control mice (7 of 7 mice) met euthanasia criteria. All HCP-treated mice survived infection (7 of 7 mice). The difference in survival between PBS-treated and HCP-treated K18-hACE2 mice was significant (log-rank; *P*=0.0001), as was weight loss on days 4–7 (T-test; *P*<0.005). C57BL/6 mice served as negative controls, these animals do not develop disease because of low affinity mouse ACE2-spike interactions. All C57BL/6 mice survived and did not exhibit weight loss. Pharyngeal swabs were collected on days 2, 4 and 6 and virus genome was detected in all infected K18-hACE2 mice at each time point, but not uninfected control mice and only in a few infected C57BL/6 mice (Fig. 3b). The difference in RNA copies was significant (*t*-test; *P*<0.05) between PBS-treated and HCP-treated on days 2 and 6, but not significant on day 4. On day 6, three mice per group were euthanized to assess infection. Lung homogenates taken on day 6–7 from PBS-treated control mice (*n*=3 time-point and *n*=7 euthanized due to disease) showed high levels of virus RNA, but viral loads from HCP-treated mice (*n*=3) were several logs lower (2.3×10^7^ copies ml^−1^ versus 1.8×10^5^ copies ml^−1^), though this was not significant (*t*-test; *P*>0.05) (Fig. 3c). Uninfected K18-hACE2 mice and infected C57BL/6 mice had minimal levels of RNA in the lungs. These findings showed that plasma from SARS-CoV-2 convalescent patients can protect K18-hACE2 against severe and lethal disease.

### Convalescent plasma reduces lung inflammation and viral load in K18-hACE2 mice

**Fig. 4.**
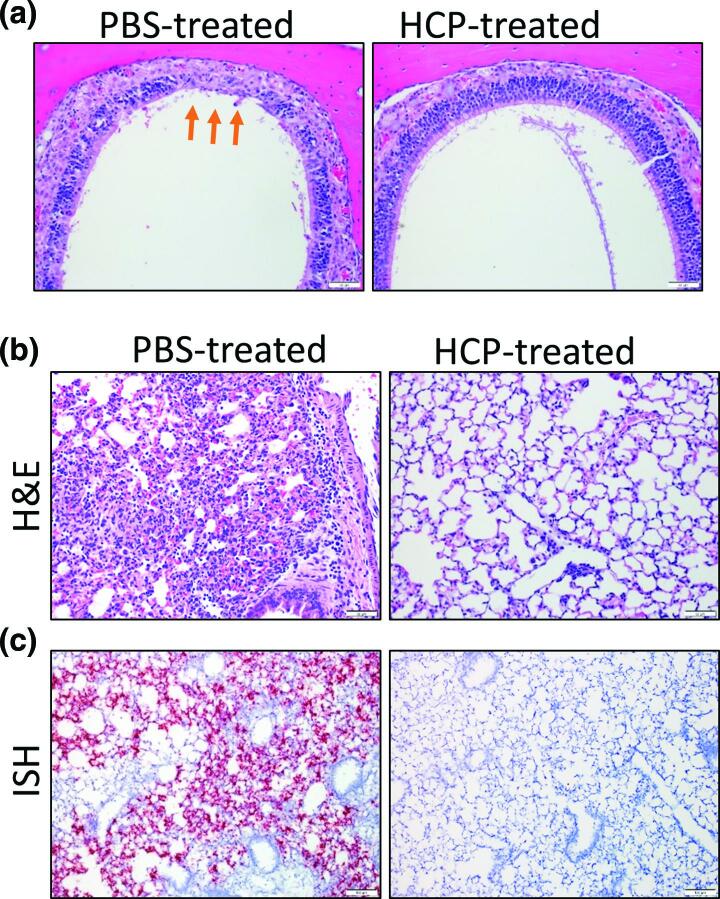
SARS-CoV-2 respiratory damage in K18-hACE2 is disrupted by HCP treatment. (a) Representative H and E staining of the rostral aspect of dorsal nasal meatus showing degeneration and atrophy of olfactory epithelium in the infected, PBS-treated animals (orange arrows). HCP-treated mice showing normal olfactory epithelium in the same region.** (b**) Representative H and E stained sections of lung in infected K18-hACE2 mice. The PBS-treated image shows an area of lung consolidation with inflamed alveolar septa and minimal adjacent perivascular inflammation and the HCP-treated image shows a lung with absence of alveolar inflammation. (**c**) Representative ISH staining showing the presence of SARS-CoV-2 RNA (red) in the lungs of infected K18-hACE2 mice. Cells were counterstained with hematoxylin. ISH staining was predominantly found in alveolar septa within the lung.

**Fig. 5.**
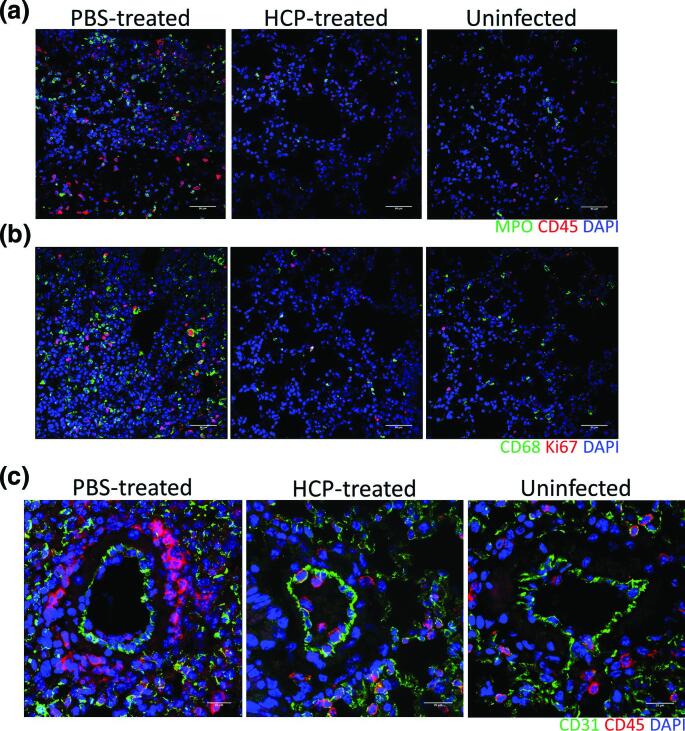
Infiltrating cells in the lungs of SARS-CoV-2 infected mice. (a) Immunofluorescence demonstrates increased number of myeloperoxidase (MPO)^+^ polymorphonuclear cells (neutrophils, eosinophils, and basophils) (green) and CD45^+^ leuckocytes (red). Nuclei are stained with DAPI (blue). (**b**) Immunofluorescence demonstrates increased number of proliferating Ki67^+^ cells (red) and CD68^+^ macrophages (green) infiltrates in the lung of infected mice in comparison with the lung of uninfected mice. Nuclei are stained with DAPI (blue). (**c**) IFA staining for CD31^+^ endothelial cells (green) and CD45^+^ leukocytes (red) in the lung of indicated mice. Nuclei are stained with DAPI (blue).

Nasal turbinates and lungs collected from K18-hACE2 mice on day 6 (*n*=3 mice per group), or at the time of euthanasia due to disease severity (*n*=6; PBS-treated group) were histologically examined. (Table S1**)**. In PBS-treated mice, the rostral aspect of dorsal nasal meatus showed degeneration and atrophy of olfactory epithelium (Fig. 4a). Two of three of the day 6 HCP-treated mice also had minimal-mild degeneration and atrophy of olfactory epithelium, a lthough it appeared less extensive in some cases (Fig. 4a). Lungs from PBS-treated mice showed damage ranging from minimal to moderate, characterized by alveolar septal mononuclear inflammation/thickening and minimal to moderate perivascular inflammation (Figs 4b and S2). Other lesions included the presence of vascular and perivascular inflammation-induced expansion of the vessel wall. Lung injury in two of three HCP-treated mice was markedly reduced with one treated animal showing largely normal lung histology with the absence of alveolar inflammation. Lung from a single HCP-treated animal exhibited peribronchial, perivascular and vascular inflammation extending into adjacent alveolar septa that was surrounded by uninflamed, normal lung. Another HCP-treated mouse had minimal mononuclear inflammation in the pulmonary interstitium surrounding bronchioles and small vessels. Viral genome was prevalent in PBS-treated mice with SARS-CoV-2 virus lung labelling, predominantly in alveolar septa (Fig. 4c). This contrasted with HCP-treated mice, where two had undetectable ISH signal, and another had minimal positive staining associated with an area of inflammation (Fig. S2a). As compared to uninfected mice (average 16.2 MPO^+^ cells and 33 CD45^+^ leukocytes per 1.25×10^5^ µm^2^ tissue area, *n*=13 areas), PBS-treated mice (average 63.5 MPO^+^ cells and 58.1 CD45^+^ leukocytes per 1.25×10^5^ µm^2^ tissue area, *P*<0.01, *n*=10 areas), but not antibody-treated mice (18.7 MPO^+^ cells and 33.5 CD45^+^ leukocytes per 1.25×10^5^ µm^2^ tissue area, *P*>0.05, *n*=13 areas), had marked increases in MPO^+^ polymorphonuclear cells (neutrophils, eosinophils, and basophils) and CD45^+^ leukocytes (Fig. 5a). Additional compared to uninfected (average 16.2 CD68^+^ macrophages and 11.2 Ki-67^+^ proliferating cells per 1.25×10^5^ µm^2^ tissue area, *n*=13 areas) HCP-treated (average 18 CD68^+^ macrophages and 11.2 Ki-67^+^ proliferating cells per 1.25×10^5^ µm^2^ tissue area, *P*>0.05, *n*=5 areas) mice, PBS-treated mice (average 75.5 CD68^+^ macrophages and 37.5 Ki-67^+^ proliferating cells per 1.25×10^5^ µm^2^ tissue area, *P*<0.01, *n*=6 areas) had higher number of CD68^+^ (macrophage) cell populations and Ki-67^+^ proliferating cells (Fig. 5b). Furthermore, as compared to uninfected control (average 16.4 CD45^+^ leukocytes per tissue area within 100 µm radius, *n*=13 areas), plasma treatment prevented perivascular CD45^+^ leukocytes infiltration in the lung, indicated by limited CD31^+^ endothelial cells/CD45^+^ leukocytes co-staining in HPC-treated (average 15.5 CD45^+^ leukocytes per tissue area within 100 µm radius, *P*>0.05, *n*=11 areas), but not PBS-treated mice (average 41.2 CD45^+^ leukocytes per tissue area within 100 µm radius, *P*<0.01, *n*=12 areas, Fig. 5c). Overall, these findings indicate that SARS-CoV-2 targeting HCP protected the lung from acute injury, limited inflammatory cell infiltration and reduced viral replication.

### Brain infection was not detected in HCP- treated K18-hACE2 mice

**Fig. 6.**
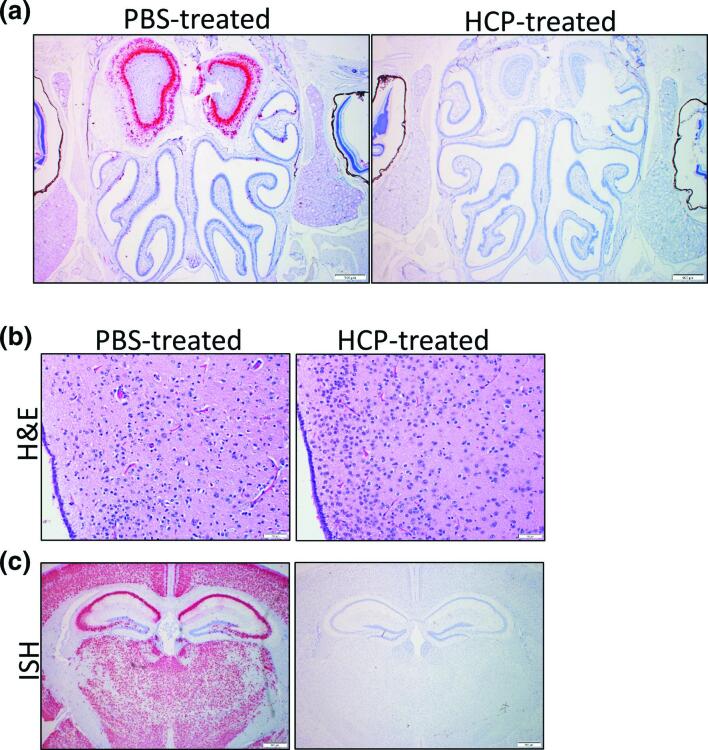
HCP-treatment prevents SARS-CoV-2 brain infection. (a) ISH labelling of SARS-CoV-2 in PBS-treated and HCP-treated mice. ISH labelling for SARS-CoV-2 in the nasal cavity and olfactory bulb. Within the olfactory bulb, a strong positive signal is present in the glomerular, external plexiform, mitral cell and internal plexiform layers. Low numbers of cells within the olfactory epithelium lining the dorsal nasal meatus have a positive ISH signal. No staining was observed in HCP-treated mice. Cells were counterstained with hematoxylin. (**b**) Representative H and E staining of the periventricular hypothalamic region showing neuronal necrosis in PBS-treated but not HCP-treated mice. (**c**) Representative ISH staining of the brain demonstrating SARS-CoV-2 labelling in neurons of the cerebral cortex, thalamus, hypothalamus, hippocampus and amygdala of a PBS-treated animal. Staining was not observed in antibody treated mice.

The majority of animals (five of six) in the challenged PBS-treated group had moderate SARS-CoV-2 genome labelling in the olfactory bulb, predominantly within the mitral cell layer, external plexiform layer and glomerular layers (Table S2). Three of challenged PBS-treated animals had histopathological findings in the olfactory bulb including microgliosis and perivascular haemorrhage. An additional two mice had neuronal vacuolation (suggestive of a degenerative change) in addition to neuronal necrosis and perivascular inflammation. Two of the challenged, PBS-treated animals did not have changes in the olfactory bulb. However, no ISH labelling nor tissue damage was observed in the olfactory bulb of HCP-treated mice (Fig. 6a).

The majority (five of six) PBS-treated mice showed signs of brain injury. This damage included meningitis, haemorrhage, vascular inflammation, perivascular inflammation, microgliosis, fibrin thrombi and neuronal necrosis (Figs 6b and S3, Table S2). One of the animals also had a neutrophilic component to the vascular/perivascular inflammation and neutrophilic inflammation in the grey matter distributed multifocally. No lesions were detected in the brains of HCP-treated mice. SARS-CoV-2 RNA was detected in most (five of six) infected PBS-treated animals. Labelling was observed in the cerebral cortex, striatum (including olfactory tubercle), thalamus, hypothalamus, hippocampus, midbrain and brainstem (pons/medulla). However, no viral RNA was observed in the brain of HCP treated mice on day 6 (*n*=3) or on day 17 (*n*=4) (Fig. 6c). These findings demonstrate that mice treated with SARS-CoV-2 targeting human plasma are protected against brain infection.

## Discussion

Antibody-based therapeutics are a major focus area of SARS-CoV-2 drug discovery [40, 41]. Several antibody products are in human trials and emergency use FDA approvals have been granted for a Regeneron cocktail immunotherapeutic (casirivimab and imdevimab). Other antibodies are being evaluated for protective efficacy in animal models, with the hamster infection model being the most widely used [34, 42, 43]. Our findings demonstrate that antibody-based therapeutics can be successfully evaluated in the K18-hACE2 system. This confirms the findings of an earlier study, which utilized a limited number of mice, where antibody was protective in the K18 model [27]. During the preparation of our manuscript, data in a pre-print article supports the use of K18-hAE2 mice for post-exposure evaluation of antibodies [44]. More specifically, that study revealed that antibody treatment, even 24 h post-challenge, can protect K18-hACE2 mice from lethal disease and that optimal protection requires functional Fc-domains. A limitation of our work here was the use of PBS as a fluid negative control and not normal human plasma, however, non-specific plasma did not protect K18-hACE mice in the prior study, further supporting our findings [27]. The advantage of the K18-hACE2 model is that, contrary to other models that only produce mild disease and limited weight loss, this model is characterized by substantial weight loss and mortality. Thus, having more obtrusive and easily captured correlates could help accelerate the evaluation of SARS-CoV-2 targeting therapeutics. Moreover, the wide availability of mouse reagents, including tools to interrogate immune responses, will permit extensive evaluation of the mechanisms of MCM action.

This study and our previous work [25] shows that 2×10^4^ p.f.u. of SARS-CoV-2 strain WA01/2020 causes severe and lethal disease in K18-hACE2 mice and this is consistent with other studies [28]. In contrast, one study reported that a log higher dose of strain WA01/2020 (10^5^) was required for uniform lethality in the K18-hACE2 system, with 10^4^ pfu producing only 35 % lethality [27]. The difference in virulence most likely is in the preparation of the virus and, more specifically, the cell lines used for propagation. The more virulent challenge stocks were produced in variants of a primate kidney epithelial cell line Vero CCL81 [28] or Vero76 cells [25]. Whereas, virus stocks tend to be more attenuated *in vivo* were produced in CALU-3, a human lung epithelial adenocarcinoma cell line [27]. It is well known that cell culture passage of virus can negatively impact host susceptibility [45]. In working with Machupo virus, we found that cell culture passage had produced a truncation of the l-segment that nullified virulence in guinea pigs while only slightly impacting replication *in vitro* [46]. Some studies report that SARS-CoV-2 cell culture passage can attenuate strains due to loss of the furin cleavage site [47]. It would be interesting to determine if propagation of SARS-CoV-2 in CALU-3 impacts virulence due to cell culture-mediated mutation or selects for quasi-species variants that have better cell culture growth potential but reduced virulence *in vivo*. This may have important implications for animal models development and could provide insight into attenuation strategies for SARS-CoV-2.

SARS-CoV-2 causes neurological sequela in at least a third of human cases including headache, confusion, anosmia meningitis, seizures and ischaemic and haemorrhagic stroke. The mechanics of neurological disease is somewhat enigmatic and it is unclear if it results from virus in the brain or from the inflammatory response to viral infection. More recent findings indicated COVID-19 survivors can develop long-term sequela (>2 month), including chronic fatigue, ongoing respiratory problems and ‘brain fog’ [15, 16]. Here we report that the K18-hACE2 model may have utility in the study of SARS-CoV-2 and persistent disease. Analysis of mice surviving the initial SARS-CoV-2 challenge on day 28 revealed potently neutralizing antibodies against SARS-CoV-2, and lungs of these mice showed varying degrees of recovery. A single mouse still had brain lesions and viral RNA. A limitation of this work is that it only examined a small number of mice (*n*=4). The original intent of that study was to produce a uniformly lethal infection, however our findings suggest adjustment of the challenge dose could increase the number of animals that develop persistent brain infection, allowing for a more thorough analysis using a larger number of animals. Other work has shown that the K18-hACE2 model can be used to study anosmia [27]. Accordingly, the K18-hACE2 system may be important in the evaluation of MCMs and may also have further use in the study of SARS-CoV-2 pathology, including neurological aspects of disease and host recovery.

## Supplementary Data

Supplementary material 1
