# Supplementary material for: Human convalescent plasma protects K18-hACE2 mice against severe respiratory disease
Source: J Gen Virol. 2021 May 7;102(5):001599. doi: 10.1099/jgv.0.001599 (PMC8295914; doi:10.1099/jgv.0.001599)
Supplement: Supplementary material 1 [file jgv-102-1599-s001.pdf]

Supplementary Materials for

Human convalescent plasma protects K18-hACE2 mice against severe respiratory disease

Joseph W. Golden*, Xiankun Zeng, Curtis R. Cline, Aura R. Garrison, Lauren E. White, Colin J. Fitzpatrick, Steven A. Kwilas, Philip A, Bowling, Jimmy O. Fiallos, Joshua L. Moore, Willie B. Sifford, Keersten M. Ricks, Eric M. Mucker, Jeffrey M. Smith and Jay W. Hooper

*Corresponding author. E-mail: joseph.w.golden.civ@mail.mil

This PDF file includes:
Supplemental Materials and Methods
Figs. S1 to S3
Tables S1 and S2

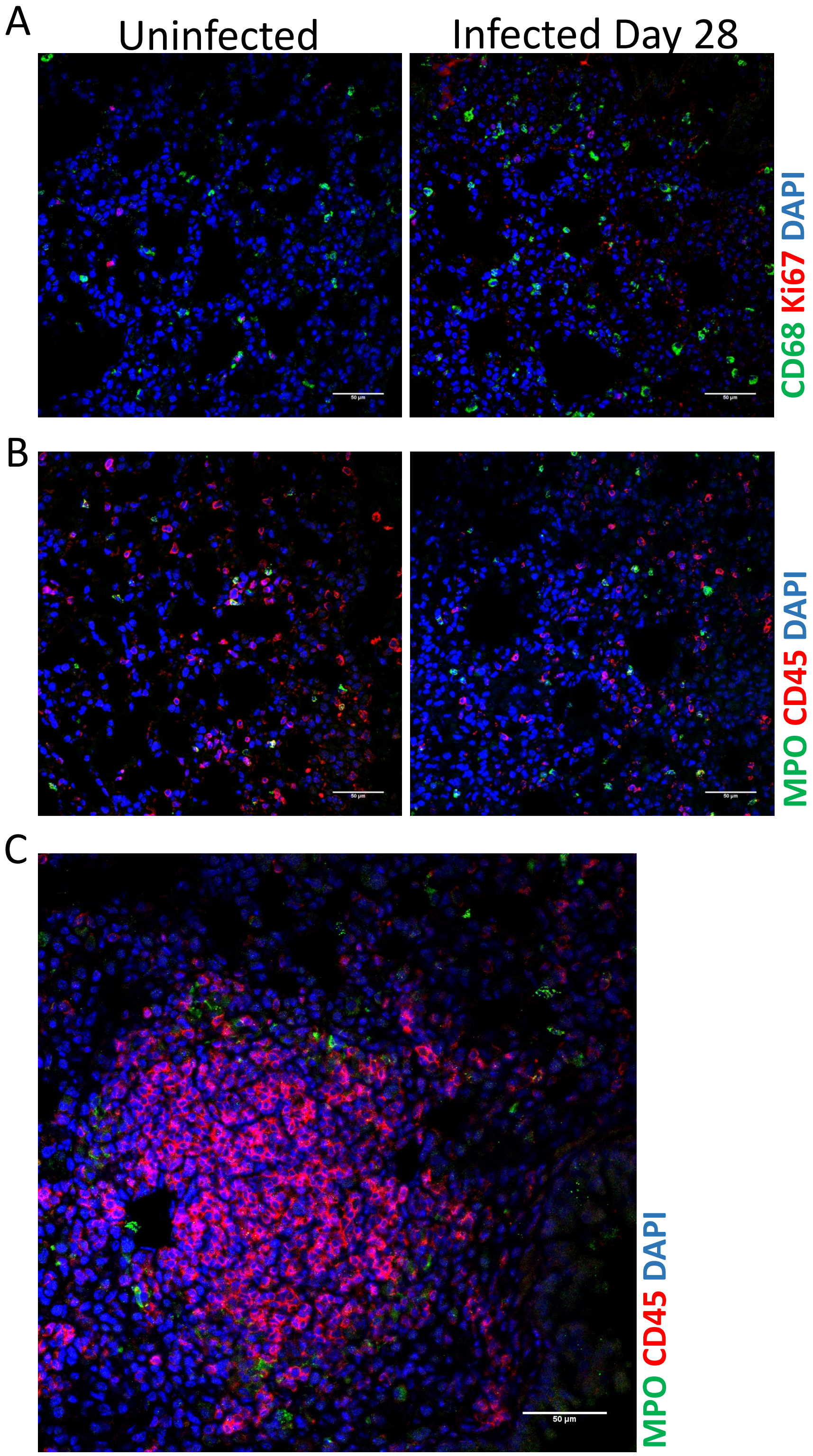

Figure S1. Immune cells in the lungs of surviving K18-Ace2 transgenic mice A-C. IFA demonstrates increased number of $CD68^+$ macrophages (A, red) in lungs of a surviving mouse in comparison with the lung of uninfected mice. No increases in MPO (B, green) or $CD45^+$ leukocytes (B, red) were observed in lung of infected mice in comparison with the lung of uninfected mice. An increase in $CD45^+$ cells (C, red) was seen in hyperplastic lymphoid tissue. But no increase in MPO^+ cells were indicated. Nuclei are stained with DAPI (blue).

Table S1. Lung lesions in SARS-CoV-2 infected K18-hACE2 mice

Organ	Microscopic Finding	n/6 untreated	Severity* untreated	n/3 treated (day 6 euth mice)	Severity* treated
Lung	Vascular inflammation in small to intermediate size vessels	5/6	Minimal - mild	1/3	Mild
	Perivascular inflammation	6/6	Minimal – moderate	2/3	Minimal - mild
	Alveolar septal inflammation / thickening	6/6	Minimal - moderate	1/3	Mild
	Alveolar accumulation of mononuclear leukocytes	4/6	Minimal – mild	1/3	Mild
	Type II pneumocyte hyperplasia	2/6	Minimal	0/3	
	Multinucleate cells (macrophages or viral syncytia)	0/6		0/3	
	Bronchial epithelial degeneration	5/6	Minimal	0/3	
	Peribronchial interstitial inflammation	0/6		2/3	Minimal - mild
	Submucosal bronchi inflammation	3/6	Minimal - moderate	0/3	
	Positive In situ hybridization (ISH) for SARS-CoV-2	6/6	Minimal - marked	1/6	Minimal
Nasal Turbinates	Degeneration, atrophy or erosion of olfactory epithelium	5/6	Minimal – mild	2/3	Minimal - mild
	Exudate within nasal meatus	1/6	Moderate	0/6	
	Positive In situ hybridization (ISH) for SARS-CoV-2	6/6	Minimal	1/3	Minimal

*Severity scores for ISH and histologic findings are based on the following: Minimal = If 10% or less of the cells in the section are immunoreactive or are affected respectively; Mild = If between 11% and 25% of the cells in the section are immunoreactive or are affected respectively; Moderate = If between 26% and 50% of the cells in the section are immunoreactive or are affected respectively; Marked = If between 51% and 79 of the cells in the section are immunoreactive or are affected respectively; Severe = If between 80% or more the cells in the section are immunoreactive or are affected respectively.

Table S2. Brain lesions in SARS-CoV-2 infected K18-hACE2 mice

Organ	Microscopic Finding	n/6 untreated	Severity* untreated	n/3 treated (day 6 euth mice)	Severity* treated
Olfactory bulb	Microgliosis	2/6	Minimal	0/3	
	Neuronal vacuolation	2/6	Minimal - mild	0/3	
	Neuronal necrosis	2/6	Minimal	0/3	
	Perivascular hemorrhage	3/6	Minimal	0/3	
	Perivascular inflammation	2/6	Minimal	0/3	
	Positive In situ hybridization (ISH) for SARS-CoV-2	5/6	Moderate	0/3	
Brain	Meningitis	4/6	Mild	0/3	
	Perivascular hemorrhage	4/6	Minimal – mild	0/3	
	Vessel inflammation	3/6	Mild - moderate	0/3	
	Perivascular inflammation	5/6	Minimal - moderate	0/3	
	Microgliosis (adjacent to vessels)	5/6	Minimal - moderate	0/3	
	Fibrin thrombi	3/6	Minimal	0/3	
	Neuronal necrosis	5/6	Minimal - mild	0/3	
	Neuronal vacuolation	4/6	Minimal	0/3	
	Neutrophilic inflammation (away from vessels)	1/6	Mild	0/3	
	Positive In situ hybridization (ISH) for SARS-CoV-2 (note 11/15 positive in the olfactory bulb)	5/6	Marked - severe	0/3	

*Severity scores for ISH and histologic findings are based on the following: Minimal = If 10% or less of the cells in the section are immunoreactive or are affected respectively; Mild = If between 11% and 25% of the cells in the section are immunoreactive or are affected respectively; Moderate = If between 26% and 50% of the cells in the section are immunoreactive or are affected respectively; Marked = If between 51% and 79 of the cells in the section are immunoreactive or are affected respectively; Severe = If between 80% or more the cells in the section are immunoreactive or are affected respectively.

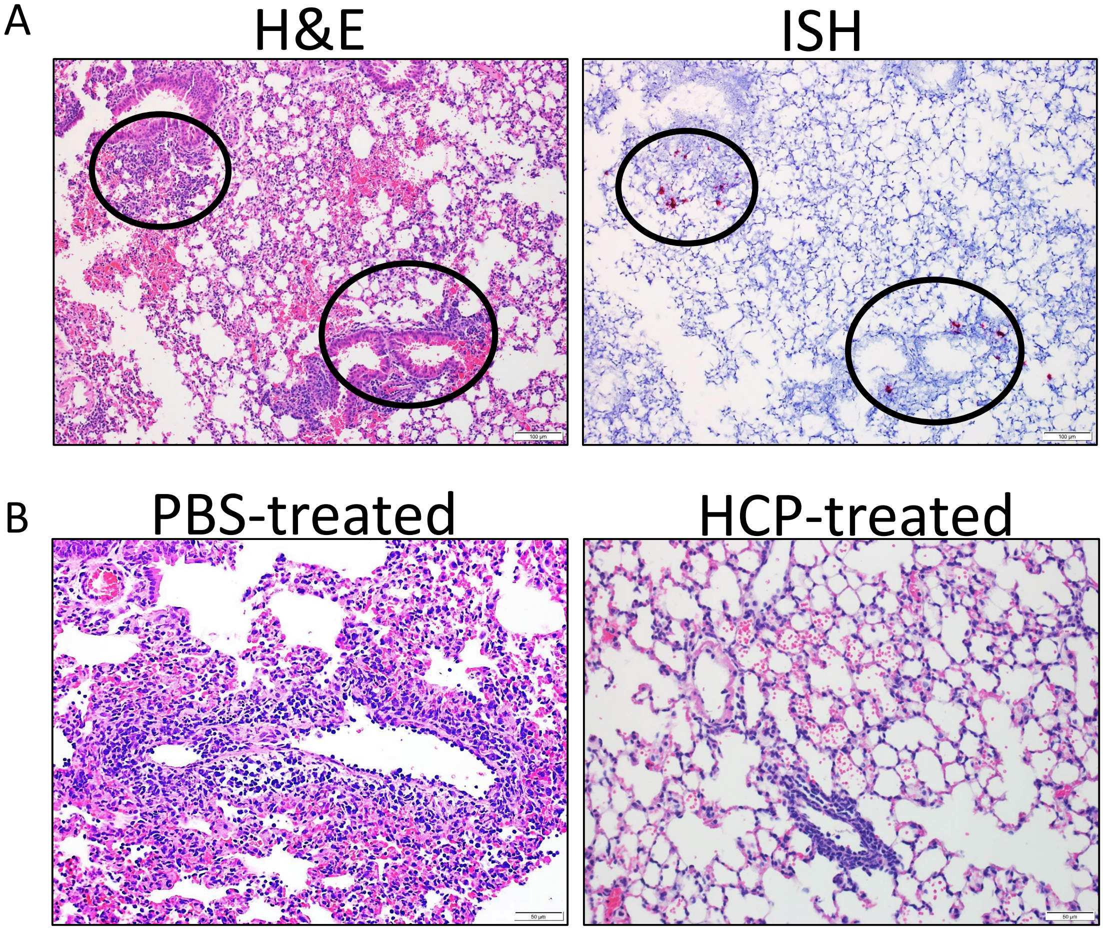

Figure S2. SARS-CoV-2 in the lungs in K18-Ace2 transgenic mice. A. H&E and ISH images from lungs of infected K18-hACE2 mice treated with HCP. ISH shows the presence of SARS-CoV-2 RNA (red). H&E shows minimal mononuclear inflammation in the pulmonary interstitium surrounding bronchioles and small vessels in the same area as the ISH staining (circles). Staining was performed in a different mice compared to Figure 4. ISH panel was were counterstained with hematoxylin. B. H&E images from other PBS- and HCP-treated mice. A minimal amount of perivascular inflammation in the vessel was detected in the HCP-treated mouse. In contrast, H&E staining of the control PBS-treated mouse shows vascular and perivascular inflammation expanding the vessel wall.

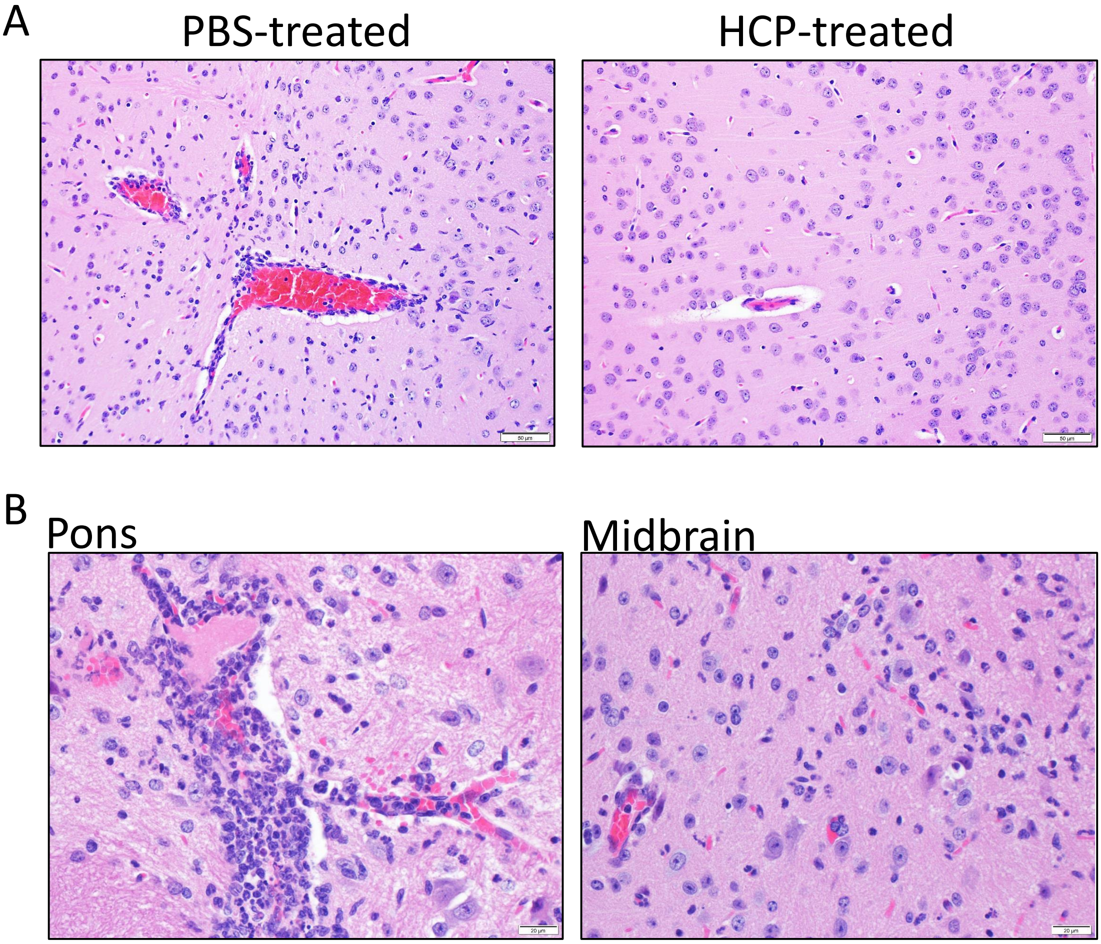

Figure S3. SARS-CoV-2 infection in K18-Ace2 transgenic mice. A. Cerebral cortex from virus challenged, PBS-treated (left panel) mouse demonstrating perivascular inflammation, congestion and microgliosis in the neuropil surrounding affected vessel. Right panel shows essentially normal cerebral cortex from a treated, virus challenged mouse corresponding to region shown in image A; note the absence of inflammation and gliosis. **B**. Vascular and perivascular inflammation in the pons of a virus challenged, untreated mouse; inflammation has a prominent neutrophilic component and a fibrin thrombus is present. Neutrophilic component was unique to this animal. Midbrain of the same animal and demonstrates neutrophils within the neuropil. The other untreated animals did not have a neutrophilic component to the CNS
